# DKK1+ tumor cells inhibited the infiltration of CCL19+ fibroblasts and plasma cells contributing to worse immunotherapy response in hepatocellular carcinoma

**DOI:** 10.1038/s41419-024-07195-3

**Published:** 2024-11-07

**Authors:** Guangyu Fan, Ruyun Gao, Tongji Xie, Lin Li, Le Tang, Xiaohong Han, Yuankai Shi

**Affiliations:** 1 Department of Medical Oncology, National Cancer Center/National Clinical Research Center for Cancer/Cancer Hospital, Chinese Academy of Medical Sciences & Peking Union Medical College, Beijing Key Laboratory of Clinical Study on Anticancer Molecular Targeted Drugs, Beijing, China; 2 Department of Pathology, National Cancer Center/National Clinical Research Center for Cancer/Cancer Hospital, Chinese Academy of Medical Sciences & Peking Union Medical College, Beijing, China; 3 Clinical Pharmacology Research Center, Peking Union Medical College Hospital, State Key Laboratory of Complex Severe and Rare Diseases, NMPA Key Laboratory for Clinical Research and Evaluation of Drug, Beijing Key Laboratory of Clinical PK & PD Investigation for Innovative Drugs, Chinese Academy of Medical Sciences & Peking Union Medical College, Beijing, China

**Keywords:** Cancer microenvironment, Tumour immunology, Immunotherapy

## Abstract

Intra-tumor immune infiltration plays a pivotal role in the interaction with tumor cells in hepatocellular carcinoma (HCC). However, its phenotype and related spatial structure remained elusive. To address these limitations, we conducted a comprehensive study combining spatial data (38,191 spots from eight samples) and single-cell data (56,022 cells from 20 samples). Our analysis revealed two distinct infiltration patterns: immune exclusion and immune activation. Plasma cells emerged as the primary cell type within intra-tumor immune clusters. Notably, we observed the co-location of CCL19+ fibroblasts with plasma cells, which secrete chemokines and promote T-cell activation and leukocyte migration. Conversely, in immune-exclusion samples, this co-location was primarily observed in the adjacent normal area. This co-localization correlated with T cell infiltration and the formation of tertiary lymphoid structures, validated by multiplex immunofluorescence conducted on twenty HCC samples. Both CCL19+ fibroblasts and plasma cells were associated with favorable survival outcomes. In an immunotherapy cohort, HCC patients who responded favorably exhibited higher infiltration of CCL19+ fibroblasts and plasma cells. Additionally, we observed the accumulation of DKK1+ tumor cells within the tumor area in immune-exclusion samples, particularly at the tumor boundary, which inhibited the infiltration of CCL19+ fibroblasts and plasma cells into the tumor area. Furthermore, in immune-exclusion samples, the SPP1 signaling pathway demonstrated the highest activity in communication between tumor and immune clusters, and CCL19-CCR7 played a pivotal role in the self-communication of immune clusters. This study elucidates immune exclusion and immune activation patterns in HCC and identifies relevant factors contributing to immune resistance.

## Introduction

Hepatocellular carcinoma (HCC) stands as the predominant subtype of primary liver cancer and ranks as the fourth leading cause of cancer-related mortality [1]. Patients with advanced-stage HCC often face negative outcomes and limited responsiveness to conventional treatments [2]. While emerging immunotherapy presents a promising treatment across various cancers, its efficacy in HCC remains limited [3–5]. Consequently, there is a pressing need for a comprehensive understanding of factors that impede immune infiltration and devising combination strategies to surmount immune resistance.

The tumor microenvironment (TME) harbors a diverse array of innate and adaptive immune cells, which can either reside within the tumor or be recruited to the site [6, 7]. These components collectively play pivotal roles in tumorigenesis, progression, metastasis, and are closely associated with the response to immunotherapy [8, 9]. Among them, intra-tumor immune infiltration emerges as a key component within the TME, interacting with tumor cells and exerting either pro-tumor or anti-tumor effects [10]. Despite its significance, studies on the TME in HCC have primarily focused on specific markers or cell types, such as PD-L1 expression and CD8 T-cell infiltration, leading to considerable variability and limited consensus [11, 12]. Moreover, the traditional description of the TME has been subjective, often relying on immunohistochemistry staining and bulk transcriptomics data without thoroughly exploring cell composition and interactions [13, 14]. Additionally, the dynamic interplay between these immune cells and tumor cells profoundly influences the immune status of the tumor, thereby impacting its response to immunotherapy [15]. A more nuanced understanding of these dynamics is essential for advancing our knowledge of HCC and developing improved therapeutic strategies targeting the TME.

Bulk RNA-seq offers a mixed expression profile of various cell types within a tissue, such as epithelial, endothelial, stromal, and immune cells. In contrast, single-cell RNA sequencing provides a more detailed understanding of transcriptional diversity at the level of individual cells, offering insights into intricate intercellular signaling networks within the TME [16, 17]. Moreover, recent advancements in spatial transcriptomics (ST) technologies have provided powerful tools to elucidate the spatial distribution of genes and uncover the TME’s composition, influencing tumor development and therapeutic responses [18]. By preserving tissue architecture, ST facilitates the examination of both molecular and cellular components of intra-tumor immune infiltration and their interactions with neighboring TME components.

In this study, we leveraged ST to characterize the phenotype of intra-tumor immune infiltration in HCC. We observed the co-location of CCL19+ fibroblasts and plasma cells in immune clusters of immuneactivation samples. Conversely, in immune exclusion samples, this co-location was primarily observed in the adjacent normal area. Both CCL19+ fibroblasts and plasma cells were associated with favorable survival. In the meanwhile, DKK1+ tumor cells accumulated in the tumor area in immune-exclusion samples, especially in the tumor boundary, inhibiting the infiltration of CCL19+ fibroblasts and plasma cells into tumor area. In conclusion, our study sheds light on the phenotype of intra-tumor immune infiltration in HCC and identifies the relevant TME factors that contributed to immune resistance.

## Materials/Subjects and Methods

### Patient samples

The FFPE tissues were obtained from patients who underwent lung resection and were diagnosed with HCC at the Cancer Hospital, Chinese Academy of Medical Sciences in Beijing, China. The collection of tissue blocks adhered to institutional ethical guidelines and was conducted following informed consent from the patients. Approval for the study protocol was obtained from the Ethics Committee of Institut Curie (No. 23/262-4004). Prior to their inclusion in the study, all patients provided written informed consent. The research was conducted in compliance with relevant ethical regulations, including the principles outlined in the Declaration of Helsinki.

### Data and materials

The single-cell datasets GSE151530, GSE149614, GSE166635, and GSE146115 were obtained from the GEO database, along with their corresponding clinical data and metadata from the original studies [16, 19–21]. For bulk-level analysis, mRNA expression data and clinical information for HCC patients were retrieved from TCGA database, accessible through UCSC Xena (http://xena.ucsc.edu/). Additionally, we incorporated ST data from GSE238264 in GEO database, focused on HCC patients treated with immunotherapy [22]. The dataset included samples from a clinical trial of neoadjuvant cabozantinib (a multi-tyrosine kinase inhibitor primarily blocking VEGF) and nivolumab (a PD-1 inhibitor), with responders and non-responders distinguished based on treatment outcomes.

### Spatial transcriptomics sequencing

FFPE human cancer tissue blocks were procured from patients, and five-micrometer FFPE sections of these samples were mounted onto IHC slides. The slides were then incubated at 42 °C for 2 hours and subsequently air-dried at room temperature. Following this, the slides underwent an additional drying step for 3 hours at 60 °C. Hematoxylin (Dako, Part number S330930-2) and Eosin (Sigma-Aldrich, Product number HT110216) were utilized for H&E staining, with staining times adjusted based on tissue type. Approximately 100 µL of 85% Glycerol (Thermofisher, Catalog number 15514011) was added, coverslips were applied, and tissue imaging was performed. To remove the coverslips, a beaker filled with Milli-Q water was employed. The Visium slide was inserted into a cassette, and 100 µL of 0.1 N HCl (Sigma-Aldrich, Product number H1758) was added to each well, followed by incubation at 42 °C for 15 minutes. After removal of the HCl, decrosslinking buffer was added, and the slide was incubated at 95 °C for 1 hour. The pre-hybridization step was conducted according to the Visium Spatial Gene Expression for FFPE reagent kit protocol (10×Genomics, User Guide CG000407 Rev C, human transcriptome Product number 1000338). 100 µL of pre-hybridization mix was added to each well and incubated at room temperature for 15 minutes. Subsequently, the pre-hybridization mix was aspirated, and 100 µL of hybridization mix was added. The Visium slide was then incubated with the hybridization mix overnight at 50 °C. For the remaining steps of library preparation, including probe ligation, probe release and extension, probe elution, and FFPE library construction, the instructions outlined in the user guide of the “Visium Spatial Gene Expression for FFPE reagent kit” (10× Genomics, User Guide CG000407 Rev C, mouse transcriptome Product number 1000339, human transcriptome Product number 1000338) were followed meticulously. The completed libraries were subsequently sequenced using the Novaseq6000 platform (Illumina). The read lengths for read 1 and read 2 were 28 base pairs and 91 base pairs, respectively.

### Pathological annotations for HE images

All spots were individually annotated by two pathologists, Lin Li and Tongji Xie. Utilizing a cell-type-specific coverage threshold of >50%, the pathologists categorized the spots into histological classes, which included normal hepatocytes, tumor cells, stromal cells, and immune cells.

### Clustering analysis of spatial transcriptomics

The ST data underwent clustering analysis using Seurat, with careful adjustment of parameters to achieve an optimal classification of cell types. To address the variance in sequencing depth across spatial spots, particularly for technical artifacts and tissue anatomy, we applied the SCTransform function, which utilizes regularized negative binomial regression to normalize molecular count data and identify high-variance features. Dimensionality reduction was conducted using principal component analysis, followed by the construction of a shared nearest neighbor graph based on the Jaccard index between spots using the first 30 dimensions. Cluster determination was carried out using the FindClusters function with a resolution of 0.8, employing SNN modularity optimization.

### Identification of malignant cells in spatial analysis

A set of immune-related signatures, including pan-immune markers (PTPRC), pan-T cell markers (CD2, CD3D, CD3E, CD3G), B cell markers (CD79A, MS4A1, CD79B), and myeloid cell markers (CD68, CD14), was employed for spot scoring. The average of these feature values was calculated as the immune score for each spot. Following clustering analysis, the cluster with the highest median immune score was identified as the reference for inferCNV analysis. Subsequently, inferCNV analysis was performed with the following parameters: cutoff = 0.1, cluster_by_groups = FALSE, denoise = TRUE, HMM = TRUE, analysis_mode = “subclusters,” tumor_subcluster_partition_method = “random_trees,” HMM_type = “i6.” The Hidden Markov Model was utilized to evaluate CNV levels within spots. To distinguish between malignant and non-malignant spots, hierarchical clustering using the inferCNV package with the random trees method divided all observation spots into 8 clusters. Spots utilized as references were labeled “reference.” In inferCNV analysis, a gene state of 3 indicated no CNV variation, a state greater than 3 indicated CNV amplification and a state less than 3 indicated CNV deletion. The CNV score for each gene was computed as the absolute value of the gene state minus 3. The cluster CNV score was determined as the sum of CNV scores for all genes. The tumor cluster was identified based on CNV scores and pathological annotations.

### Differential expression analysis and gene set enrichment analysis

DEG analysis within each cluster was performed using the FindAllMarkers function of the Seurat package, with parameters set as min.pct = 0.1 and logfc.threshold = 0.25. To explore the biological functions of the identified DEGs in each cluster, gene set enrichment analyses (GSEA) were conducted using the R package fgsea. This analysis encompassed the evaluation of enrichment in cancer hallmarks, Biological Process Gene Ontology (GO), and Kyoto Encyclopedia of Genes and Genomes (KEGG) gene sets.

### Dimension reduction and clustering analysis for single-cell data

The top 2000 most variable genes were identified using the FindVariableFeatures function and subsequently employed for principal component analysis within the Seurat package. To mitigate batch effects within the datasets, we applied the Harmony algorithm from the Harmony R package before conducting the clustering analysis. Cell subtypes were identified through the FindNeighbors and FindCluster functions. Cells were annotated using curated markers, including epithelial cells (EPCAM, KRT8, KRT19), fibroblasts (COL1A1, COL1A2, DCN), endothelial cells (PLVAP, VWF, PECAM1), T cells (CD3D, CD3E, TRAC), B cells (MS4A1, CD79A), and myeloid cells (CD14, CD163, CD68, FCGR3A).

### Transcription factor analysis

The DoRothEA database is a valuable gene set resource comprising TFs and their interactions with target genes, enabling the inference of TF activity from gene expression data [23]. Interactions with confidence levels A, B, and C are included in our study. To estimate the activities of the regulons, we utilized the run_viper function, integrating the VIPER algorithm with the DoRothEA package. This approach allowed us to estimate TF activities from the Dorothea regulons effectively. Additionally, we employed the “FindAllMarkers” function to identify the top TFs for each cluster, sorting them based on log2 fold change.

### MIF

The first MIF panel, comprising panCK (abcam, ab234297), CCL19 (abmart PU622961), DCN (abcam ab268048), DKK1 (abcam, ab109416), IGHG1 (abcam, ab109489) and the second panel, consisting of CCL19 (abmart PU622961), IGHG1 (abcam, ab109489), CD3D (abcam, ab109531), CD8A (abcam ab268048) were conducted following the manufacturer’s instructions provided by Akoya’s Multiple IHC Kit. Tumor tissues obtained from surgeries were fixed in formalin and embedded in paraffin. Immunofluorescence staining was performed according to standard procedures using the Leica BOND RX platform (YTAn00042). Initially, paraffin sections underwent repair and blocking steps, followed by incubation with primary antibodies targeting the panel components. Subsequent to additional incubation for 30 minutes to 1 hour at room temperature, the sections were treated with secondary antibodies for 10 minutes, followed by the appropriate opal fluorophore (620, 480, 570, 690, 520, and 780, respectively; Alphaxbio, AXT37100041) reagent at room temperature for 10 minutes. Finally, the paraffin sections were stained with DAPI (9:125 dilution) for 5 minutes at room temperature and subjected to standard analysis by Halo Link software (Indica Labs).

### IHC analysis

A total of 70 HCC samples underwent IHC analysis. Following dewaxing, the slices were incubated with specific primary antibodies (DKK1 (abcam, ab109416) and CCL19 (abmart PU622961)) at 4 °C overnight, succeeded by incubation with biotinylated secondary antibody (Proteintech, Wuhan, China) at room temperature for 1 hour. Positive staining was visualized using DAB chromogenic reagent, and each section was subsequently counterstained with hematoxylin. Each sample received a score based on the intensity of staining (0 = no staining; 1 = weak staining; 2 = moderate staining; and 3 = strong staining) and the proportion of stained cells (0 = 0%; 1 = 1–25%; 2 = 25–50%; 3 = 50–75%; 4 = 75–100%). The final score was computed as the product of the staining intensity and positive area score, ranging from 0 to 12. The IHC results of tissues were independently reviewed by two experienced pathologists who were blinded to the clinical parameters.

### Survival analysis

For survival analyses, patient samples were stratified into two groups, high and low, based on gene expression levels. This categorization was performed using the surv_cutpoint function within the R survminer package. To evaluate the association between gene expression and overall survival time, we employed the survival and survminer packages. The log-rank test was utilized to determine the difference between the survival curves of the two groups. Subsequently, Kaplan-Meier survival curves were created using the ggsurvplot function to illustrate the survival outcomes of the two expression groups.

### Cell-Cell communication analysis

The CellChat R package was employed to infer, analyze, and visualize intercellular communication interactions between cell subsets [24]. Initially, we identified overexpressed ligands or receptors in each cell type and inferred communication probabilities by calculating all ligand and receptor interactions associated with each signaling pathway. Subsequently, the cell-cell communication network was constructed using the netAnalysis_signalingRole_scatter function. Signaling groups based on functional or structural similarity were identified using the “computeNetSimilarity” function. Further analyses included the calculation of outgoing and incoming signaling patterns of cells using the “netAnalysis_signalingRole_heatmap” function. Additionally, ligand-receptor pairs involved in signaling between cells were identified and visualized using the “netVisual_bubble” function.

### Statistical analysis

The Mann-Whitney U test was utilized to analyze the differences between the two groups, while Spearman’s correlation test was employed to assess the correlations between two variables. A significance threshold of 0.05 (two-tailed) was applied to determine statistical significance.

## Results

### The identification of malignant cells in ST data

**Fig. 1:**
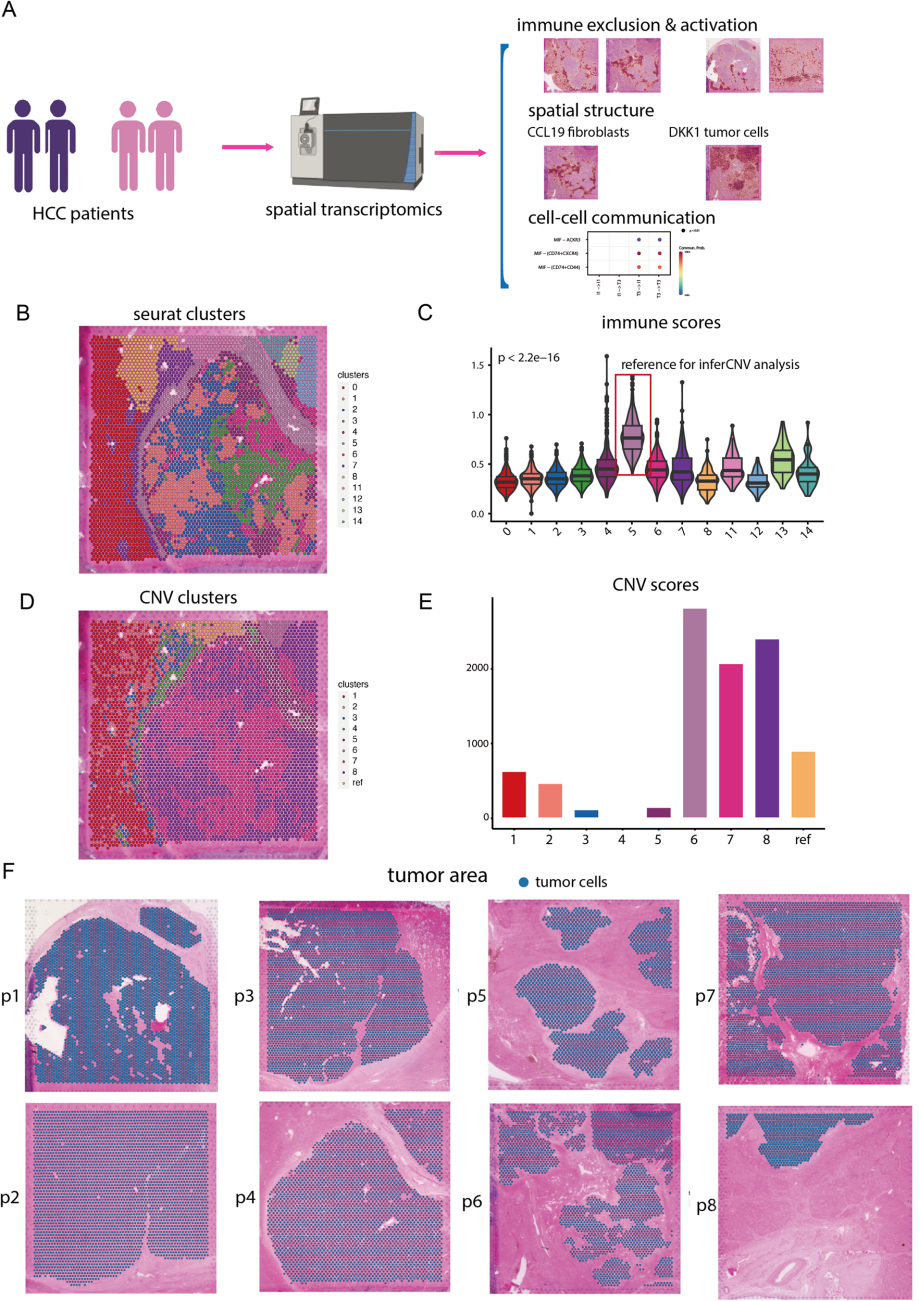
The identification of malignant cells in spatial transcriptomics (ST) data. **A** The workflow depicting the study design. **B** Clustering of all spots in patient 4 (p4) into 14 distinct clusters. **C** Distribution of immune score in the 14 clusters. **D** Hierarchical clustering assigning all spots in p4, except the reference cluster, into eight clusters. **E** Bar charts showing the distribution of copy number variation (CNV) score in the nine clusters. **F** The plot depicted the identified tumor cells in eight tumor samples.

The study design was illustrated in Fig. 1A, depicting the workflow. To explore the spatial organization of HCC, spatial transcriptomic sequencing was conducted using tumor tissue sections from eight HCC patients. Transcriptomics data were obtained, with a median of 4900 spots for all samples. The median number of genes per spot across all samples was approximately 6000, with the percentage of mitochondrial genes across all samples below 5% (Fig. S1). The detailed quality information of eight ST samples can be found in Table S1.

Similar to the challenges encountered in single-cell data analysis, identifying malignant cells based solely on gene expression patterns in ST data is challenging, particularly in distinguishing between malignant and normal epithelial cells. To address this complexity, inferCNV analysis was performed to specifically delineate malignant cells from other cell types based on their copy number variation (CNV) patterns. This process involved two clustering phases.

The first phase aimed to identify reference cells for the inferCNV pipeline, which would be used to infer CNV patterns of malignant cells. Initially, all spots were segmented into smaller clusters based on gene expression patterns. The “immune score” was calculated for each spot using a series of immune-related signatures, including pan-immune markers (PTPRC), pan-T cell markers (CD2, CD3D, CD3E, CD3G), B cell markers (CD79A, MS4A1, CD79B), and myeloid cell markers (CD68, CD14), representing the average immune infiltration within each spot. The cluster with the highest immune score was then selected as the reference for the inferCNV analysis. For instance, in patient 4 (p4), all spots were divided into 14 distinct clusters, with cluster 5 exhibiting the highest immune score and thus designated as the reference cluster for inferCNV analysis (refer to Fig. 1B, C).

In the second clustering phase, the goal was to differentiate malignant cells from other cell types based on CNV patterns. Hierarchical clustering, employing tree partitioning in the R package inferCNV, assigned all spots (except the reference cluster) into eight clusters (Fig. 1D). Clusters 6, 7, and 8, characterized by extremely high CNV scores, were identified as malignant clusters, while the remaining clusters showed significantly lower CNV scores (Fig. 1E). These annotations were validated by consulting two independent pathologists who analyzed the histology information from hematoxylin-eosin staining (HE). Clusters 6, 7, and 8 corresponded to scattered tumor areas, while the other clusters primarily consisted of normal epithelial cells, fibroblasts, and mixed immune cells. Subsequently, we applied the same pipeline to other samples and identified the tumor areas in each sample (illustrated in Fig. 1F). The tumor cells identified were all within the tumor areas annotated by the HE histology information.

### Plasma cell were the dominate cell type in the intra-tumor immune infiltration

**Fig. 2:**
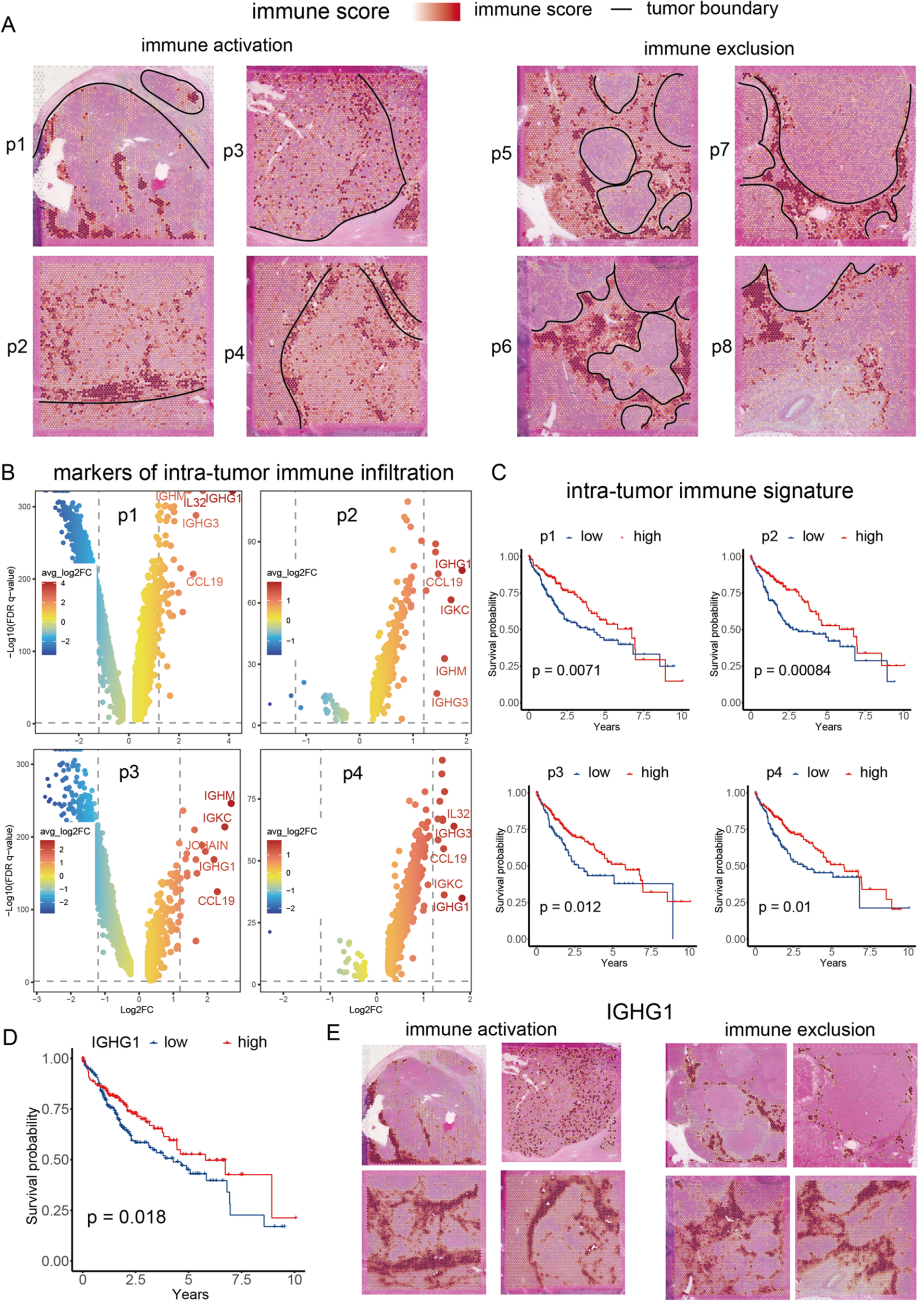
Plasma cell were the dominate cell type in the intra-tumor immune infiltration. **A** The plot depicted the tumor boundary and the distribution of immune score in eight hepatocellular carcinoma (HCC) samples. **B** The differentially expressed genes (DEGs) of the immune clusters in p1 to p4, respectively. **C** The role of infiltrated immune signature (top 50 DEGs) of p1 to p4 in patients’ survival, respectively. **D** IGHG1 as the plasma cell marker showed the favourable role in HCC patient’ survival. **E** Distribution of the plasma cell marker IGHG1 in eight HCC samples.

To explore the landscape of immune infiltration in HCC, we evaluated the immune score in each sample, which reflects the average level of immune infiltration, encompassing T cells, B cells, and myeloid cells (Fig. 2A). Notably, four samples (p5 to p8) exhibited immune exclusion from the tumor area, while the remaining four samples (p1 to p4) demonstrated significant immune activation, characterized by intra-tumor immune infiltration clusters. Furthermore, all samples displayed a noticeable accumulation of immune cells along the tumor boundary.

Given previous indications of intra-tumor immune components interacting with malignant cells and exerting both pro-tumor and anti-tumor effects, we delved into the specific cell composition of these immune clusters in the four samples (p1 to p4). Using the cloupe software provided by 10X Genomics, we manually isolated the immune clusters in each sample and identified their respective differentially expressed genes (DEGs) (Fig. 2B). Notably, all four samples exhibited elevated expression of markers associated with plasma cells (IGHA1, IGHG1, IGHG3, and JCHAIN). Recent studies have linked increased plasma cell signatures to improved overall survival (OS) in patients treated with immunotherapy, based on transcriptomic data in various cancers, including non-small cell lung cancer, sarcoma, melanoma, and renal cell carcinoma [25, 26]. Additionally, the expression of chemokines such as CCL19, IL32, and CCL21 was notably high in these immune clusters, indicating their role in promoting the immune response.

To assess the impact of these immune infiltration clusters on survival outcomes, we utilized the top 50 DEGs from these clusters in each sample, as outlined in Table S2. In a bulk-level transcriptomics cohort from The Cancer Genome Atlas (TCGA), patients exhibiting a higher immune activation signature across four samples demonstrated prolonged OS (Fig. 2C). Concurrently, the expression of IGHG1, the plasma cell marker, exhibited a favorable association with survival among HCC patients, consistent with the immune activation signature (Fig. 2D). We subsequently examined the expression patterns of IGHG1 in the spatial context. In immune activation samples, the distribution of IGHG1 corresponded predominantly with the immune activation signature (Fig. 2E). Notably, IGHG1 demonstrated high expression levels at the tumor boundary of immune exclusion samples, suggesting a potential mechanism wherein tumor cells impede the infiltration of plasma cells.

### CCL19+ fibroblasts co-located with plasma cells in the intra-tumor immune infiltration

**Fig. 3:**
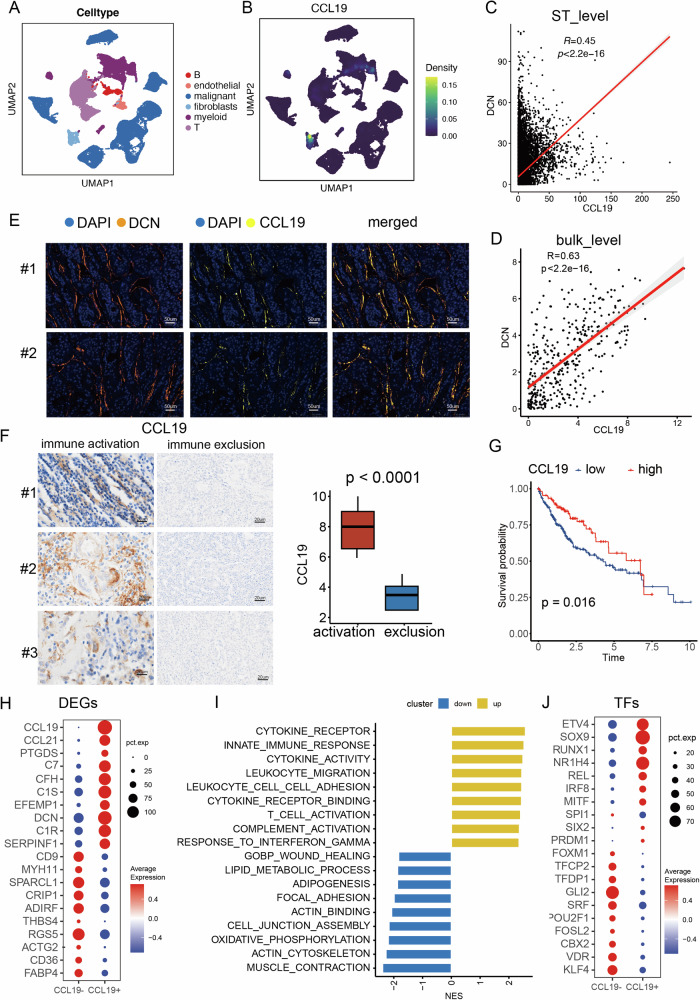
CCL19+ fibroblasts accumulated in the intra-tumor immune infiltration. **A** The single-cell dataset was divided into six main clusters: epithelial cells, myeloid cells, fibroblasts, endothelial cells, T cells, and B cells. **B** Distribution of CCL19 in single-cell dataset. **C** The plot displayed the correlation between CCL19 and DCN in the spatial transcriptomics data. **D** The plot displayed the correlation between CCL19 and DCN in the bulk transcriptomics data. **E** The multiplex immunofluorescence (MIF) perfomed in 20 hepatocellular carcinoma (HCC) patients demonstrated the co-location between CCL19 and DCN. **F** Immunohistochemistry (IHC) was performed on these samples to assess CCL19 expressiono in immune activation and immune exclusion samples. **G** HCC patients with high CCL19 levels had longer overall survival (OS) time. **H** The top 10 upregulated genes in CCL19+ and CCL19- fibroblasts. **I** Bar chart displaying the up-regulated and down-regulated pathways in CCL19+ fibroblasts. **J** Expression patterns of the most varied transcription factors (TFs) in CCL19+ fibroblasts.

CCL19 exhibited high expression levels within intra-tumor immune infiltration. To accurately identify the cellular source of CCL19, we collected multiple single-cell datasets (including GSE151530, GSE149614, GSE166635, and GSE146115) and classified all cells into six primary clusters: epithelial cells, myeloid cells, fibroblasts, endothelial cells, T cells, and B cells [16, 19–21] (Fig. 3A). The distribution of CCL19 was predominantly observed in fibroblasts, suggesting its characteristic role as a chemokine derived from fibroblasts (Fig. 3B). A recent study has highlighted that CCL19 released from fibroblasts can attract dendritic cells and T lymphocytes [27]. Additionally, local administration of CCL19-expressing mesenchymal stem cells has been shown to enhance the therapeutic efficacy of anti-PD-L1 antibodies by facilitating the infiltration of immune cells [21].

To validate this finding, we assessed the correlation between CCL19 and the fibroblast marker DCN, which reached 0.45 in the intra-tumor immune infiltration spots (Fig. 3C). In the bulk transcriptomics data, this correlation increased to 0.63, further confirming CCL19 as a chemokine released from fibroblasts (Fig. 3D). To validate CCL19 at the protein level, we conducted multiplex immunofluorescence (MIF) analysis on samples from 20 HCC patients, using DCN to annotate fibroblasts. CCL19 demonstrated high expression levels around DCN+ fibroblasts, indicating its presence as a soluble chemokine released from fibroblasts (Fig. 3E). To clarify, we have already collected formalin-fixed paraffin-embedded (FFPE) samples to further investigate this relationship. Specifically, we analyzed ten samples with confirmed immune activation and ten samples with immune exclusion. Immunohistochemistry (IHC) was performed on these samples to assess CCL19 expression. Our results consistently demonstrated higher expression of CCL19 in the immune activation samples compared to the immune-exclusion samples (Fig. 3F). In a bulk-level transcriptomics cohort, patients with higher levels of CCL19 exhibited significantly longer OS, further supporting its role in promoting the immune response (Fig. 3G).

We then further elucidated the phenotype of CCL19+ fibroblasts. First, by comparing the CCL19+ and CCL19- fibroblasts, we compiled a list of DEGs (Fig. 3H). Among these DEGs, CCL21 could illicit immunotherapy response and controls extensive migratory events in the immune system [28]. Notably, C7, CFH, and C1S were three complement-related genes found to be highly expressed in CCL19+ fibroblasts, with all of them having associations with promoting innate immune response [29, 30]. Conversely, CD36 and FABP4, the lipid metabolism-related proteins, exhibited down-regulation in CCL19+ fibroblasts [31]. Additionally, muscle-related contractile-related genes including ACTG2, RGS5, and MYH11 were suppressed in CCL19+ fibroblasts.

Pathway analysis unveiled increased immune activities in CCL19+ fibroblasts (Fig. 3I). Specifically, chemokine activities were significantly enhanced in CCL19+ fibroblasts, encompassing both the secretion and reception of chemokines, potentially facilitating immune cell recruitment to inflammatory sites. Moreover, CCL19+ fibroblasts exhibited heightened T cell activation and leukocyte migration. Conversely, lipid metabolic processes and adipogenesis were suppressed in CCL19+ fibroblasts. Additionally, pathways related to smooth muscle contraction, such as actin cytoskeleton organization, actin binding, and muscle contraction, were also downregulated in CCL19+ fibroblasts.

Furthermore, we investigated the role of transcription factors (TFs) in promoting the phenotype of CCL19+ fibroblasts (Fig. 3J). Remarkable findings included enhanced regulon activities of ETV4, which is associated with the cell cycle and the Wnt/β-catenin signaling pathway [32]. Additionally, SOX9 exhibited high expression levels, capable of inducing extracellular matrix, growth factor, and inflammatory gene expression, thereby facilitating matrix deposition [33, 34]. Furthermore, RUNX1 displayed increased activities and serves as a key driver of fibroblast states, linking to the promotion of macrophage-myofibroblast transition in non-small-cell lung carcinoma [35, 36].

### The co-location of plasma cells and CCL19+ fibroblasts conferred a therapeutic benefit to patients undergoing immunotherapy for HCC

**Fig. 4:**
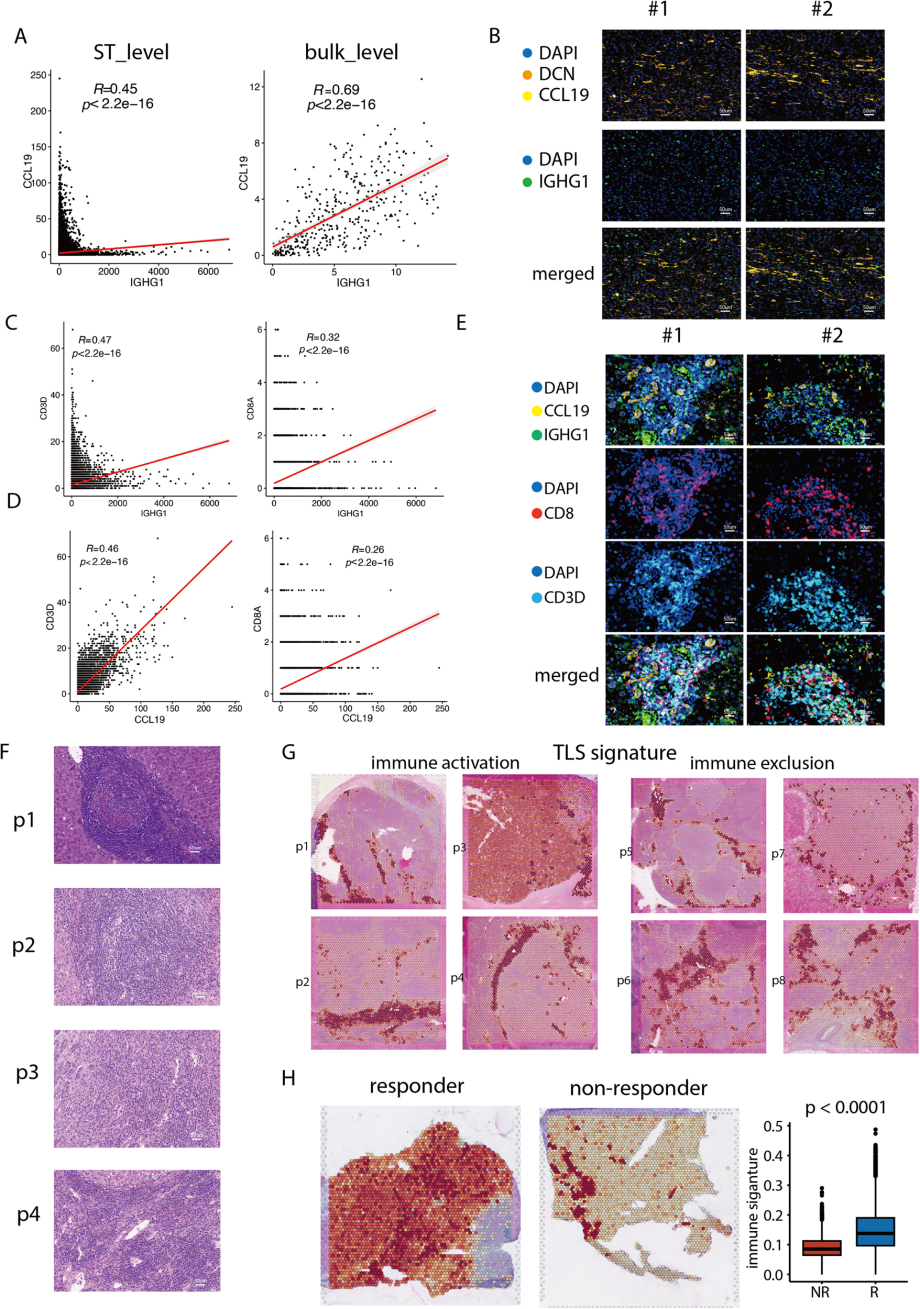
The co-location of plasma cells and CCL19+ fibroblasts conferred a therapeutic benefit to patients undergoing immunotherapy for hepatocellular carcinoma (HCC). **A** The correlation between CCL19 and the plasma cell marker IGHG1 at spatial transcriptomics (ST) and bulk transcriptomics level. **B** The multiplex immunofluorescence (MIF) performed on 20 HCC patients demonstrated the co-location of IGHG1+ plasma cells and CCL19+ fibroblasts. **C** The correlation between IGHG1 and the T cells marker CD3D at ST and bulk transcriptomics level. **D** The correlation between CCL19 and the T cells marker CD3D at ST and bulk transcriptomics level. **E** The MIF images demonstrated the co-location of IGHG1+ plasma cells, CCL19+ fibroblasts and CD3D+ T cells. **F** The hematoxylin and eosin (HE) images of immune infiltration in samples (p1 to p4). **G** The spatial distribution of tertiary lymphoid structures (TLS) score. **H** The distribution of immune activation signature (including CD79A, IGHG1, IGHG2, IGHG3, and CCL19) in a cohort of HCC patients treated with immunotherapy.

To validate the co-localization of plasma cells and CCL19+ fibroblasts, we evaluated the correlation between CCL19 and the plasma cell marker IGHG1, which reached 0.45 at the ST level (Fig. 4A). In the bulk transcriptomics data, this correlation increased to 0.69, further confirming the strong association between plasma cells and CCL19+ fibroblasts (Fig. 4A). To validate the association at the protein level, we conducted MIF analysis on samples from 20 HCC patients, utilizing panCK to annotate tumor cells, DCN to annotate fibroblasts, and CD79 to annotate B cells. Our observations revealed IGHG1+ plasma cells scattered within the tumor area, with CCL19 exhibiting high expression levels around these plasma cells, suggesting its role as a supportive component for plasma cells (Fig. 4B).

Given previous reports indicating B cells’ role in promoting T cell formation, we then assessed the correlation between plasma cells/CCL19+ fibroblasts and T cells. Both the plasma cell marker IGHG1 and CCL19 exhibited significant associations with the T cell marker CD3D at both the ST and bulk transcriptomics levels (Fig. 4C, D). To validate these associations at the protein level, we conducted MIF analysis on samples from 20 HCC patients. Our observations revealed CD3D+ T cells accumulating in clusters within the tumor area and tumor boundary, with IGHG1+ plasma cells and CCL19+ fibroblasts scattered within these T cell clusters (Fig. 4E).

Subsequently, we examined the hematoxylin and eosin (HE) images of immune activation samples (p1 to p4). The high immune score corresponding to the accumulation of immune cells resembled tertiary lymphoid structures (TLS) (Fig. 4F). We compiled a gene list of TLS from a published study that utilized spatial transcriptomics to examine TLS in renal cell carcinoma [37]. This signature included 12 immunoglobulin genes (IGHA1, IGHG1, IGHG2, IGHG3, IGHG4, IGHGP, IGHM, IGKC, IGLC1, IGLC2, IGLC3, and JCHAIN), 5 B cells markers (CD79A, FCRL5, MZB1, SSR4, and XBP1), 2 T cells markers (TRBC2 and IL7R), 2 fibroblasts markers (CXCL12, LUM), 2 complement protein coding genes (C1QA and C7), and CD52, APOE, PTLP, PTGDS, PIM2, and DERL3 genes. The TLS score exhibited a similar spatial distribution as plasma cells and CCL19+ fibroblasts, showing low expression levels in immune exclusion tumor areas (Fig. 4G).

Additionally, we incorporated ST data from a recent study focusing on HCC patients treated with immunotherapy [22]. This dataset included samples from a clinical trial of neoadjuvant cabozantinib (a multi-tyrosine kinase inhibitor that primarily blocks VEGF) and nivolumab (a PD-1 inhibitor), with responders and non-responders distinguished based on treatment outcomes. Notably, responders exhibited a higher immune activation signature (including CD79A, IGHG1, IGHG2, IGHG3, and CCL19) compared to non-responders, further reinforcing our analysis and emphasizing the potential relevance of plasma cells and CCL19+ fibroblasts in immunotherapeutic responses (Fig. 4H).

### DKK1 inhibited the infiltration of intra-tumor infiltration in HCC

Tumor cells had been reported to remodel the immune components in the TME to facilitate immune evasion, tumour growth and metastasis, and therapeutic resistance. Therefore, we further investigated the phenotype of the tumor cells in samples with distinct intra-tumor immune infiltration to uncover potential tumor-immune interactions as the potential targets for immunotherapy in HCC.

**Fig. 5:**
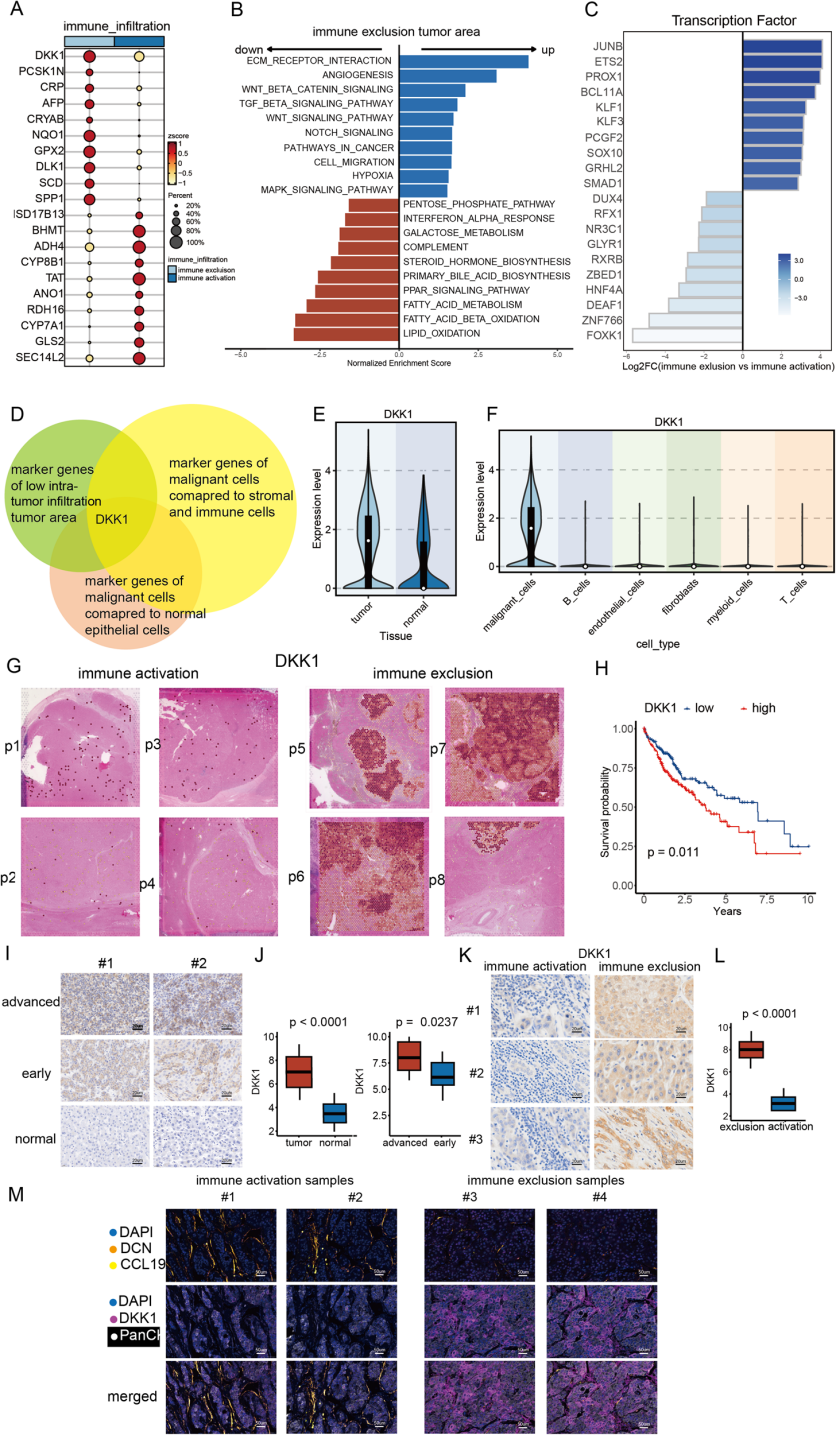
DKK1 inhibited the infiltration of intra-tumor infiltration in hepatocellular carcinoma (HCC). **A** The differentially expressed genes (DEGs) of tumor cells in samples with distinct immune infiltration patterns. **B** Pathway analysis unveiled distinct biological activities in two types of tumor areas. **C** Expression patterns of the most varied transcription factors (TFs) in two types of tumor areas. **D** DKK1 was identified as the potential tumor marker correlating with immune exclusion. **E** DKK1 exhibited elevated expression in tumor cells compared to normal epithelial cells. **F** DKK1 exhibited elevated expression in tumor cells compared to immune cells and stromal cells. **G** The spatial expression patterns of DKK1 in two immune infiltration types of samples. **H** Patients with high DKK1 levels had shorter survival time. **I** Immunohistochemistry images of DKK1 in our cohort consisting of 70 HCC patients. **J** Boxplots displaying the distribution of DKK1 in normal, early and advanced stage samples. **K** Immunohistochemistry images of DKK1 in immune exclusion and immune activation samples. **L** Boxplots displaying the distribution of DKK1 in immune exclusion and immune activation samples (**M**).

Firstly, we computed the DEGs within the tumor area across samples exhibiting distinct patterns of immune infiltration (Fig. 5A and Table S3). Among these DEGs, DKK1 emerged as a crucial factor secreted by cancer stem cells [38]. DKK1’s promotion of differentiation is vital for the metastatic outgrowth of disseminated tumor cells [38]. Furthermore, NQO1 was a protective antioxidant agent and a versatile cytoprotective agent that regulates oxidative stresses of chromatin-binding proteins, thereby mitigating DNA damage in cancer cells [39]. Additionally, GPX2, an antioxidant enzyme, played a significant role in tumor progression across various cancers by promoting metastasis through phenotypic and metabolic reprogramming [40].

Pathway analysis revealed distinct biological activities in two types of tumor areas (Fig. 5B). In the immune exclusion tumor area, there was an increase in epithelial-to-mesenchymal transition (EMT) activities, angiogenesis, and hypoxia. Additionally, the well-known immunosuppressive TGFB pathway was enhanced, along with the WNT signaling pathway and WNT beta-catenin signaling. Conversely, in the immune activation tumor area, there was an enhancement of immune response-related pathways, including complement activation and interferon alpha response. Furthermore, many metabolism-related pathways, such as fatty acid metabolism, lipid oxidation, and bile acid biosynthesis, were heightened in the tumor area with immune activation.

Moreover, we investigated the role of TFs in promoting the immune exclusion phenotype. Findings revealed heightened regulon activities of JUNB in the immune exclusion tumor area, which is implicated in tumorigenesis by regulating cell proliferation, differentiation, senescence, and metastasis [41, 42]. Additionally, ETS2, which mediates the activation of downstream of RAS/MAPK signaling, exhibited increased regulon activities in the immune exclusion area [43]. Previous studies identified ETS2 and JUNB could regulate the transition through the partial EMT state. Overexpression of these regulators predicted a poor clinical outcome, and their elimination readily abolished TGF-β-induced EMT [44].

We proceeded to identify tumor-specific genes that hindered immune infiltration and imposed an immunosuppressive effect on the TME. Initially, we isolated marker genes distinct to tumor cells in contrast to immune cells and normal epithelial cells, employing criteria of avg_log2FC > 0.25 and p-value < 0.05, respectively (Fig. 5D). By intersecting these tumor marker genes with the top 50 DEGs of the immune exclusion tumor area, we pinpointed DKK1 as meeting the criteria while exhibiting the highest expression level (Fig. 5D). Notably, DKK1 manifested increased expression in tumor cells relative to immune cells and normal epithelial cells (Fig. 5E, F).

Subsequently, we analyzed the spatial expression patterns of DKK1 in samples with distinct levels of immune infiltration (Fig. 5G). DKK1 predominantly manifested expression in tumor cells as opposed to normal epithelial cells and immune/stromal cells, aligning with our findings from single-cell analysis. In immune exclusion samples, DKK1 exhibited notable expression within tumor areas, whereas in samples displaying immune activation, its expression levels were markedly lower. Additionally, in a cohort of bulk-level transcriptomics, patients with elevated DKK1 expression experienced significantly shorter OS (Fig. 5H). Furthermore, we conducted IHC on samples from 70 HCC patients to delve into the clinical significance of DKK1. DKK1 displayed heightened expression in tumor cells compared to normal epithelial cells (Fig. 5I, J). Moreover, advanced HCC patients exhibited higher DKK1 expression levels compared to those in the early stages (Fig. 5I, J). In addition, we have already collected FFPE samples to further investigate the relationship between DKK1 and immune status. Specifically, we analyzed ten samples with confirmed immune activation and ten samples with immune exclusion. IHC was performed on these samples to assess DKK1 expression. Our results consistently demonstrated higher expression of DKK1 in the immune activation samples compared to the immune-exclusion samples (Fig. 5K, L). We have conducted additional co-staining of CCL19 and DKK1 in immune-exclusion samples to further confirm our conclusion (Fig. 5M). The new data demonstrate that DKK1 is highly expressed in the tumor area of immune-exclusion samples, with DCN+CCL19+ fibroblasts excluded from the tumor core. In contrast, in immune activation samples, DKK1 expression was low, with a higher presence of DCN+CCL19+ fibroblasts.

DKK1 is expressed at low levels in liver cancer cell lines. To investigate the role of the key molecule DKK1, we constructed DKK1-overexpressing liver cancer cell lines, hepa1-6 and H22. We successfully constructed a lentiviral vector containing the mouse DKK1 sequence, lenti-EF1a-MouseDKK1-PGK-Puro (Fig. S1). The Mouse DKK1 lentivirus was then packaged and used to infect hepa1-6 and H22 cells. Finally, we successfully established DKK1-overexpressing hepa1-6 and H22 stable cell lines. To confirm the overexpression of DKK1, we performed ELISA to detect DKK1 in the cell culture supernatants. We found that DKK1 protein levels were significantly higher in DKK1+ hepa1-6 and DKK1+ H22 cells compared to the previous cell lines and the negative control groups, verifying the construction of DKK1-overexpressing stable cell lines (Fig. S2A).

We then conducted preliminary functional experiments to explore the role of DKK1 in liver cancer cells. The CCK8 cell proliferation assay revealed that cell viability was higher in DKK1+ hepa1-6 and DKK1+ H22 cells compared to the previous cell lines (Fig. S2B). Similarly, the colony formation assay showed enhanced colony-forming ability in DKK1+ H22 cells (Fig. S2C). Furthermore, Transwell assays indicated increased migration and invasion capabilities in DKK1+ hepa1-6 cells compared to the controls (Fig. S2D). These results demonstrated the potential pro-tumor function of DKK1.

### Cell-cell communication involved in immune exclusion

In immune exclusion samples, DKK1 was highly expressed in the tumor area, where plasma cells and CCL19+ fibroblasts were excluded and situated along the tumor boundary. We hypothesized that DKK1+ tumor cells inhibited the infiltration of immune components into the tumor area. Therefore, we initiated an investigation into the cell-cell communication between the tumor area and neighboring immune area in sample p6, aiming to unveil interaction patterns contributing to immune exclusion.

**Fig. 6:**
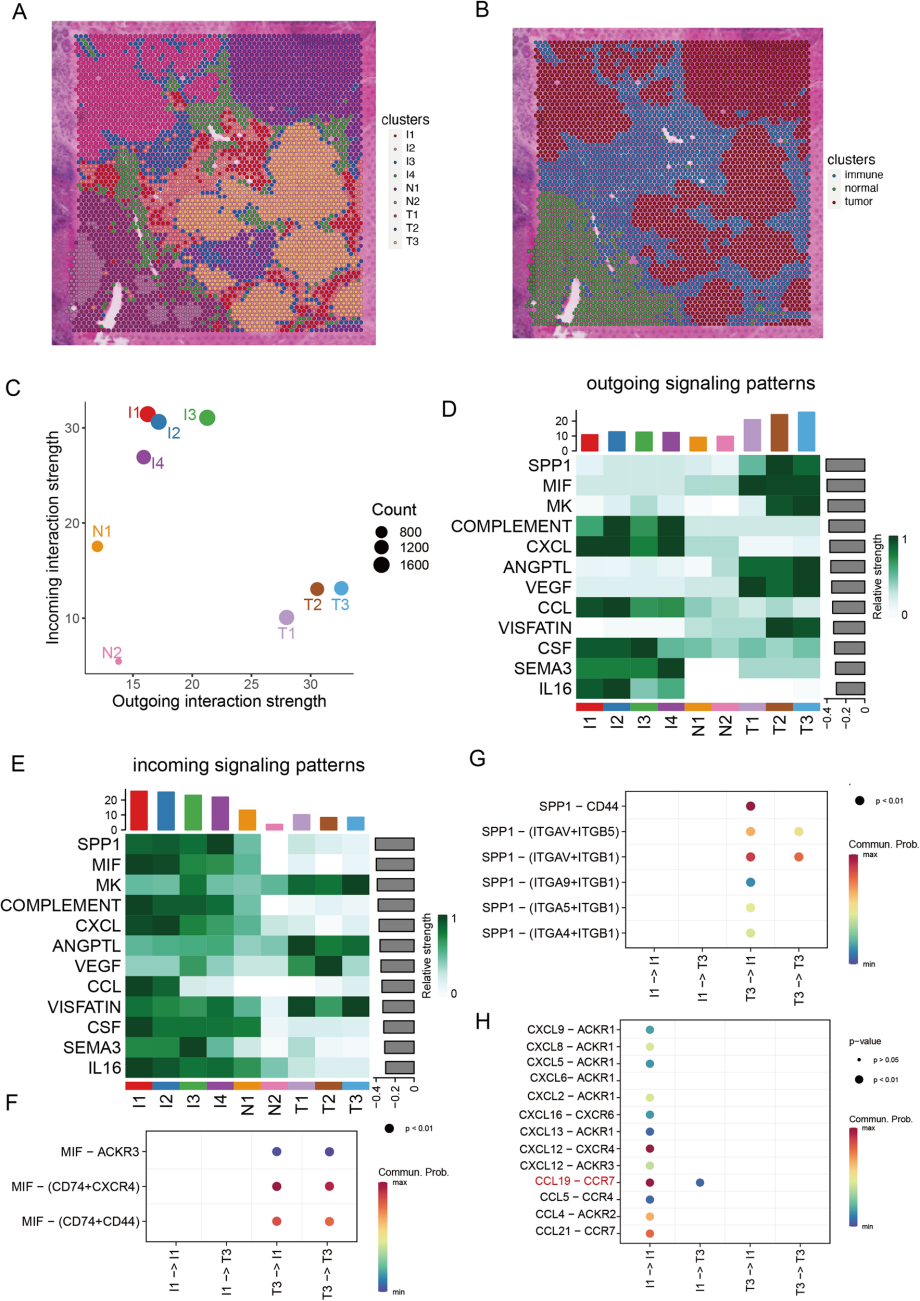
Cell-cell communication involved in immune exclusion. **A** All spots in p6 were divided into nine clusters, including four immune clusters (I1 to I4), two normal hepatocyte clusters (N1 to N2), and three tumor clusters (T1 to T3). **B** The spatial distribution of immune, tumor and normal clusters. **C** The intensity of cell-cell communication within all clusters. **D** Overview of the outgoing signaling pathways. **E** Overview of the incoming signaling pathways. **F** The specific cell-cell interactions in SPP1 signaling pathway among the tumor and immune clusters. **G** The specific cell-cell interactions in MIF signaling pathway among the tumor and immune clusters. **H** The specific cell-cell interactions in CXCL and CCL signaling pathway among the tumor and immune clusters.

All spots in p6 were divided into nine clusters, including four immune clusters (I1 to I4), two normal hepatocyte clusters (N1 to N2), and three tumor clusters (T1 to T3) (Fig. 6A). The four immune clusters encircled the tumor clusters, while the two normal clusters were positioned slightly further away (Fig. 6B). Initially, we examined the intensity of cell-cell communication within all clusters. The three tumor clusters exhibited the highest level of outgoing interactions, whereas the four immune clusters displayed the highest level of incoming interactions (Fig. 6C). These observations suggested the pivotal role of tumor cells in reshaping the tumor microenvironment towards an immune exclusion state.

Next, we analyzed the outgoing and incoming signaling pathways, revealing distinct patterns between the tumor area and normal area. The tumor area activated various proliferation and metastasis-related signaling pathways (such as MK, SPP1, SEMA3, MIF, and VEGF) and immunosuppression-related signaling pathway (GALECTIN) (Fig. 6D). On the other hand, the immune clusters were the primary recipients of signaling from the tumor clusters, whereas the normal area activated various immune-related signaling pathways (including COMPLEMENT, CCL, CXCL, and IL16), which were not received by tumor clusters (Fig. 6D, E).

Subsequently, we delved into the specific cell-cell interactions among the tumor and immune clusters. Our analysis identified the SPP1 pathway as the primary interaction between tumor and immune clusters. To simplify the presentation of specific signaling pathways, we utilized the T3 in the tumor clusters with the highest outgoing signaling activities and I1 in the immune clusters with the highest incoming signaling activities for further analysis. Specifically, SPP1 released from tumor cells interacted with CD44, ITGAV, ITGA5, ITGB1, and ITGB5 on immune clusters (Fig. 6F). Additionally, MIF released from DKK1+ tumor cells activated CD74, CXCR4, and CD44 on immune clusters (Fig. 6G). Both SPP1 and MIF are known contributors to critical cancer hallmarks, including cell growth, survival, metastasis, migration, and angiogenesis. As for the immune clusters, the chemokine-related pathways were significantly active in self-communication rather than communication with tumor clusters. We observed high interaction activities within the CXCL family (including CXCL9, CXCL12, and CXCL8) and CCL family (including CCL19, CCL4, CCL5, and CCL21) (Fig. 6H). It’s noteworthy that CCL19 activated CCR7 in the self-communication of immune clusters, indicating its role in promoting and maintaining immune infiltration.

## Discussion

Intra-tumor immune infiltration plays a crucial role in modulating antitumor immune responses, with its impact on the TME being highly intricate and contingent upon various cell components [45]. In this study, we utilized ST to elucidate the phenotype of intra-tumor immune infiltration in HCC. Our analysis unveiled the co-localization of CCL19+ fibroblasts and plasma cells within the tumor area of samples exhibiting high intra-tumor immune infiltration. Remarkably, both CCL19+ fibroblasts and plasma cells were associated with favorable survival outcomes. Conversely, DKK1+ tumor cells were observed to accumulate within the tumor area in immune exclusion samples, particularly at the tumor boundary. This accumulation impeded the infiltration of CCL19+ fibroblasts and plasma cells into the tumor area. In summary, our findings shed light on the intra-tumor immune infiltration in HCC and underscore the role of DKK1+ tumor cells in hindering the infiltration of CCL19+ fibroblasts and plasma cells, thereby contributing to immune resistance.

The initial observations in HCC revealed distinct patterns of immune infiltration, with some tumors exhibiting immune activation while others displayed immune exclusion. In our study, we found that B cells, particularly plasma cells, were the dominant cell type in intra-tumor immune infiltration. B cells have been linked to improved cancer outcomes, particularly when present in association with tertiary lymphoid structures, a finding consistent with our results [46]. They contribute to durable immune responses through mechanisms such as antibody-dependent cellular cytotoxicity, antigen presentation, and active participation in immune processes [47, 48]. Studies have demonstrated that the presence of B cells in tumor tissues correlates with enhanced response to anti-PD-1 therapy in patients with gastric cancer [26]. Additionally, B cell infiltration has been associated with improved survival outcomes following PD-1 inhibition in HNSCC [49]. Furthermore, increased stromal B cell density in pre-treatment biopsy samples of recurrent/metastatic HNSCC identifies a subgroup of PD-L1 expressors with better survival outcomes after anti-PD-1 treatment [49].

Subsequently, we observed the accumulation of CCL19+ fibroblasts within the plasma cell+ intra-tumor immune infiltration. CCL19, a chemokine renowned for its role in leukocyte recruitment and migration, holds significant importance in immune function, particularly in orchestrating the movement of T and B cells within lymphoid organs [50]. Notably, CCL19 has been found to enhance CD8+ T cell responses, thereby aiding the immune system in effectively targeting and eliminating tumor cells [51]. Studies have revealed that low CCL19 expression is associated with unfavorable outcomes in cancers such as small cell lung cancer and follicular lymphoma [52, 53]. Moreover, the CCR7 chemokine axis, which encompasses CCL21 and CCL19, has emerged as a promising target for cancer immunotherapy, demonstrating potential as a target for therapeutic intervention [54, 55]. Our study further unveiled that the co-localization of plasma cells and CCL19+ fibroblasts conferred a therapeutic benefit to patients undergoing immunotherapy for HCC. Research has demonstrated that CCL19-producing fibroblastic stromal cells can restrain lung carcinoma growth by promoting local antitumor T-cell responses [27]. Furthermore, strategies aimed at enhancing CCL19 expression, such as IL-7 and CCL19 expression in chimeric antigen receptor (CAR) T cells, have been explored [56]. These approaches aim to enhance immune cell infiltration and CAR-T cell survival within the tumor microenvironment, potentially augmenting the efficacy of CAR-T cell therapy against cancer.

Furthermore, our study unveiled that DKK1+ tumor cells amassed within the tumor region in immune exclusion samples, particularly proximal to the tumor boundary. This accumulation impeded the infiltration of CCL19+ fibroblasts and plasma cells into the tumor area. DKK1 plays a multifaceted role in cancer progression, particularly in promoting tumor growth, metastasis, and immunosuppression. Moreover, DKK1 has been implicated in hepatocellular carcinoma tumorigenesis by activating the Wnt/β-catenin signaling pathway [57]. Additionally, DKK1 has been demonstrated to regulate the accumulation and function of myeloid-derived suppressor cells in the tumor microenvironment, contributing to immunosuppression in cancer [58]. In the context of metastasis, DKK1 promotes the differentiation of disseminated tumor cells, which is pivotal for their outgrowth at metastatic sites [38]. Conversely, inhibiting DKK1 has been shown to reduce the metastatic burden by inducing dormancy in metastatic cells [38]. DKK1 inhibitors have demonstrated efficacy in inhibiting metastasis, suggesting a potential therapeutic strategy for metastatic cancer [59]. These findings underscore the diverse roles of DKK1 in cancer progression and highlight its potential as a therapeutic target for metastatic disease and immunotherapy resistance.

In comparison to previous research, our study presents several notable strengths. It stands as the first to comprehensively characterize intra-tumor infiltration patterns in HCC and examine their spatial organization. We successfully identified and validated the co-location of CCL19+ fibroblasts and plasma cells, thereby facilitating the identification of immune activation components within the TME. With over 10,000 cells analyzed in single-cell and 38,000 spots examined across eight spatial transcriptomic samples, our study achieves both breadth and depth in portraying the immune infiltration phenotype of HCC. However, it’s important to acknowledge certain limitations. Comprehensive experimental validations are necessary to interpret the role of DKK1+ tumor cells in impeding the infiltration of CCL19+ fibroblasts and plasma cells into the tumor area within the context of the immune response.

This study demonstrated the characterization of intra-tumor infiltration patterns in HCC and identified DKK1+ tumor cells impeding the infiltration of CCL19+ fibroblasts and plasma cells into the tumor area that induced immunosuppressive TME.

## Supplementary information

supplementary materials

## Data Availability

The raw sequence data reported in this paper have been deposited in the Genome Sequence Archive in the National Genomics Data Center, China National Center for Bioinformation/Beijing Institute of Genomics, Chinese Academy of Sciences (GSA:HRA006757).
