# Supplementary material for: DKK1+ tumor cells inhibited the infiltration of CCL19+ fibroblasts and plasma cells contributing to worse immunotherapy response in hepatocellular carcinoma
Source: Cell Death Dis. 2024 Nov 7;15(11):797. doi: 10.1038/s41419-024-07195-3 (PMC11541906; doi:10.1038/s41419-024-07195-3)
Supplement: Supplementary file 1 — supplementary materials [file 41419_2024_7195_MOESM1_ESM.pdf]

SUPPLEMENTARY INFORMATION

MATERIALS AND METHODS

Cell Culture

The human hepatocellular carcinoma (HCC) cell lines Hepa1-6 and H22 were purchased from Genomeditech (China). The Hepa1-6 cell line was cultured in Dulbecco's Modified Eagle's Medium (DMEM, SH30022.01, Cytiva, USA), while the H22 cell line was maintained in RPMI-1640 (SH30809.01, Cytiva, USA), both supplemented with 10% fetal bovine serum (FBS, 10091-148, Gibco, USA), 100 U/ml penicillin, and 100 mg/ml streptomycin. All cell culture was conducted at 37 °C in a humidified incubator with 5% CO2. The authenticity of these cell lines was confirmed by short tandem repeats (STRs) DNA profiling.

Construction of Lentivirus and Stable Cell Lines

To investigate the role of DKK1, we constructed a lentiviral vector containing the mouse DKK1 sequence, designated as lenti-EF1a-MouseDKK1-PGK-Puro (Figure S1). Plasmid construction followed standard molecular cloning procedures, and the resulting constructs were confirmed by DNA sequencing. An empty vector was utilized as a negative control. Lentivirus production and subsequent cell infection were performed in accordance with the protocol recommended by Genomeditech. Specifically, the lentiviral plasmid was co-transfected into HEK-293T cells along with packaging plasmids and a transfection reagent (GM easy™ Lentiviral Mix, HGTG-06, Genomeditech, China; HG Transgene™ Reagent, GMLCP-10, Genomeditech, China).

After 10-12 hours of transfection, enhancing buffer was added, and after an additional 8 hours, the medium was replaced with fresh culture medium. The cells were cultured for an additional 48 hours, after which the supernatant containing lentiviral particles was collected and concentrated to obtain a high-titer lentiviral solution. The viral titer was determined and quantified in HEK-293T cells, and the virus was subsequently stored at -80 °C.

The packaged Mouse_DKK1 lentivirus was used to infect Hepa1-6 and H22 cells, generating stable Mouse_DKK1 Hepa1-6 and Mouse_DKK1 H22 cell lines. Seventy-two hours post-infection, the cells were subjected to selection with puromycin at a lethal dose of 6 μg/ml for two cycles, followed by maintenance at a concentration of 3 μg/ml for at least 2 weeks. The selected cell pools were used for further experimental analysis.

Enzyme-Linked Immunosorbent Assay (ELISA)

Supernatants from cultured cells were collected to measure DKK1 production using an enzyme-linked immunosorbent assay (ELISA) kit (KE10033, ProteinTech, USA) in accordance with the manufacturer's instructions. All assays were performed in triplicate and repeated three times under independent conditions to ensure reproducibility.

Cell Viability Assay

We employed the Cell Counting Kit-8 (CCK8) assay (Dojindo Laboratories, Japan) to

assess cell proliferation. Hepa1-6, DKK1+ Hepa1-6, H22, and DKK1+ H22 cell lines were digested, collected, counted, and seeded into 96-well plates at a density of 2.5× 10^3 cells per well in a volume of 100 μL medium containing 10% FBS. Cell viability was assessed at 0, 24, 48, 72, and 96 hours post-seeding according to the manufacturer's instructions. Briefly, 10 μL of CCK-8 reagent was added to 90 μL of serum-free medium to create a working solution, of which 100 μL was added per well, and incubation was carried out for 3 hours.

Colony Forming Assay

Given the relatively weak colony-forming ability of Hepa1-6 and H22 cells, 5000 cells per well were seeded into 6-well plates and cultured for 2 weeks with fresh medium replenished every 3 days. After the incubation period, the medium was removed, and the plates were washed with PBS. Cells were fixed with 4% paraformaldehyde for 20 minutes, washed again with PBS, and stained with 0.1% crystal violet solution for 20 minutes. Excess stain was removed by rinsing with water, and the plates were allowed to air-dry at room temperature.

Transwell Migration Assay

For the transwell migration assay, 1×10^5 cells of each line (Hepa1-6, DKK1+ Hepa1-6, H22, and DKK1+ H22) in 200 μL of serum-free medium were added to the upper chambers of a transwell plate. The lower chambers were filled with 600 μL of

medium containing 10% FBS serving as a chemoattractant. After 8 hours of incubation, non-migratory cells on the upper surface of the chambers were removed using a cotton swab. Migrated cells were then fixed with 4% paraformaldehyde and stained with 1% crystal violet. Fields were randomly selected for observation and analysis under a microscope.

Transwell Cell Invasion Assay

For the transwell invasion assay, Matrigel (Corning, USA) was diluted 1:8 with serum-free medium, and 80 μL of the diluted matrigel was used to coat the transwell filters. The filters were then incubated for 90 minutes at 37°C and subsequently hydrated with 200 μL of serum-free medium for 30 minutes at 37°C. Following this preparation, 1×10^5 cells of each line (Hepa1-6, DKK1+ Hepa1-6, H22, and DKK1+ H22) suspended in 200 μL of serum-free medium were added to the upper chamber. The lower chambers were filled with 600 μL of medium containing 10% FBS to serve as a chemoattractant. After a 24-hour incubation period, the cells were processed as described in the migration assay, with fixation in 4% paraformaldehyde and staining with 1% crystal violet. Fields were randomly selected for observation and analysis under a microscope.

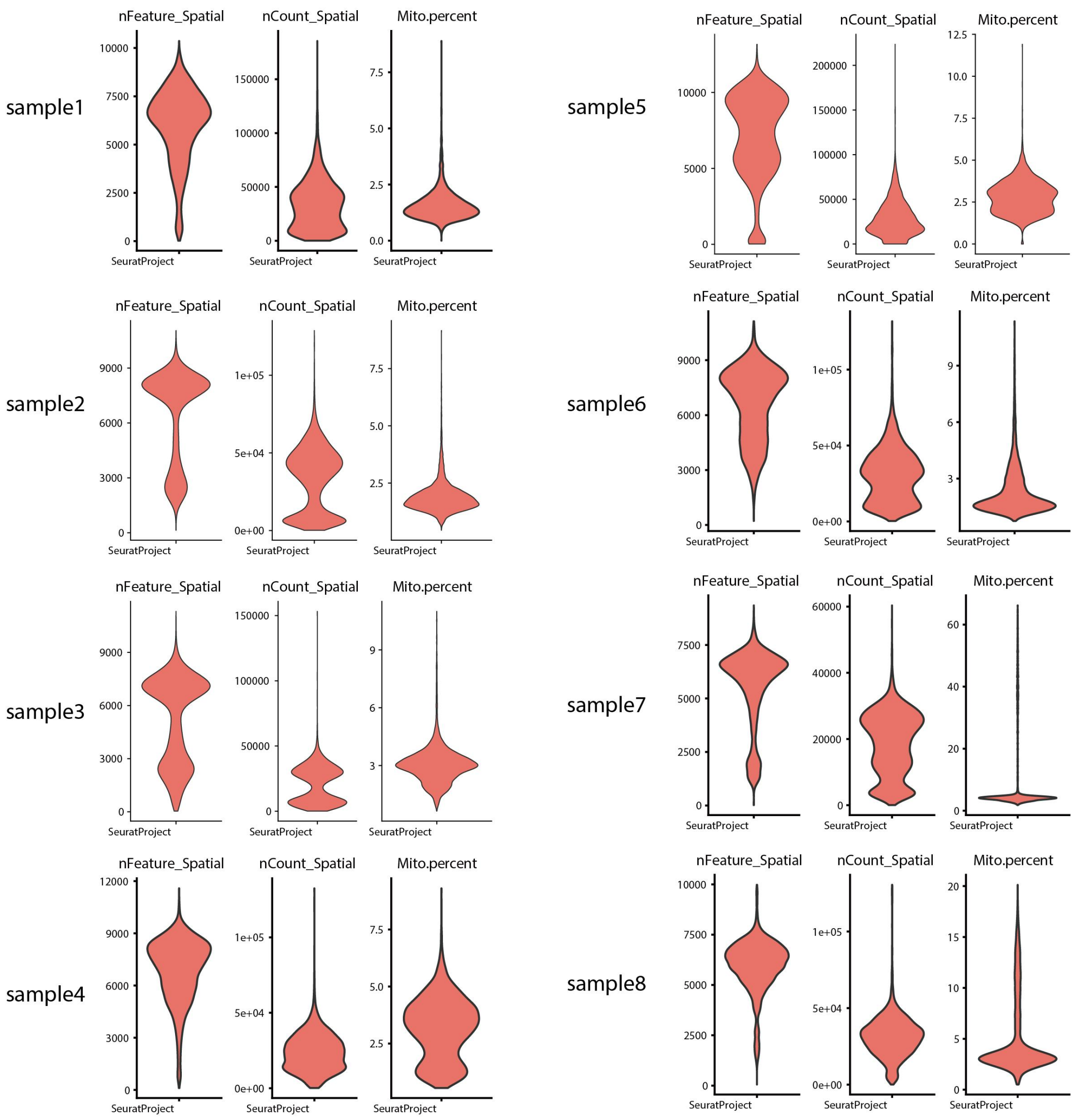

Figure S1: The violin plots of nFeature, nCount, and the mitochondrial gene percentage of eight ST samples.

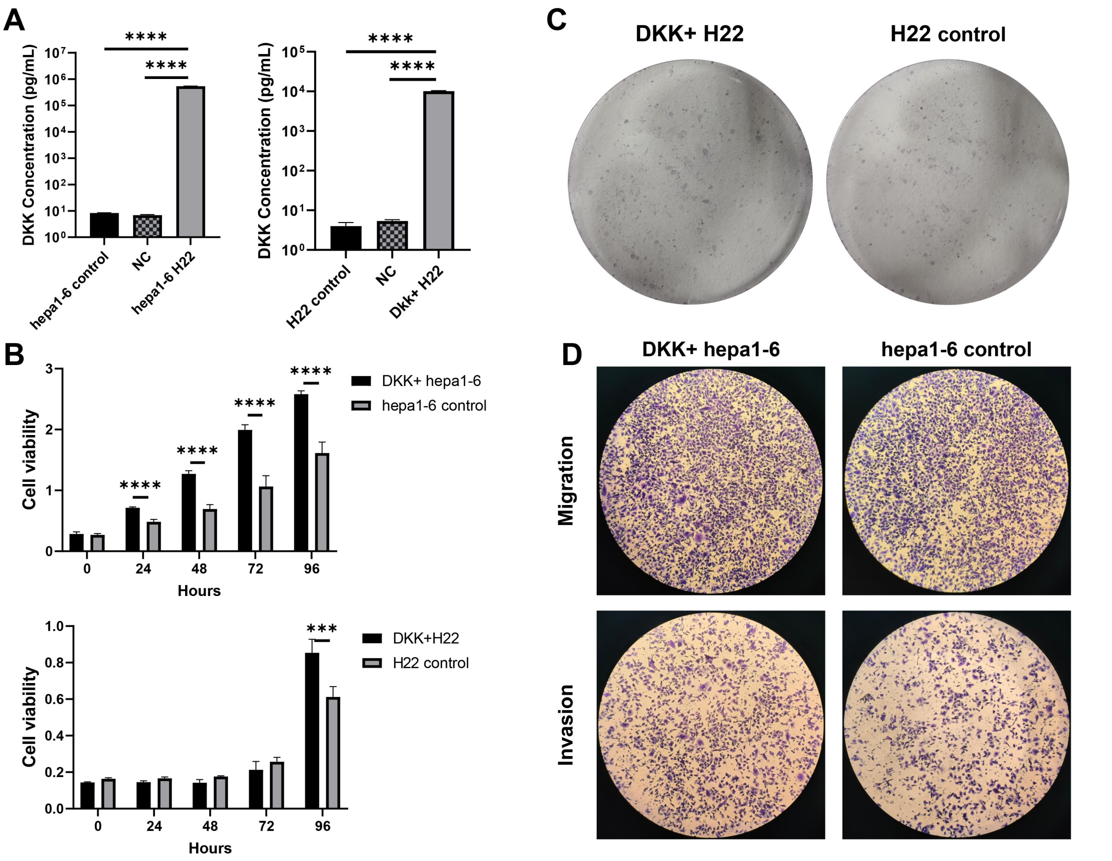

FigureS2. (A) DKK1 protein levels were significantly higher in DKK1+ hepa1-6 and DKK1+ H22 cells compared to the previous cell lines and the negative control groups. (B) The CCK8 cell proliferation assay revealed that cell viability was higher in DKK1+ hepa1-6 and DKK1+ H22 cells compared to the previous cell lines. (C) Similarly, the colony formation assay showed enhanced colony-forming ability in DKK1+ H22 cells. (D) Transwell assays indicated increased migration and invasion capabilities in DKK1+ hepa1-6 cells compared to the controls.

Table S1: The The detailed quality information of eight ST samples.

sample	age	gender	stage	Number of Spots Under Tissue	Mean Reads per Spot	Mean Reads Under Tissue per Spot	Fraction of Spots Under Tissue	Reads Mapped to Probe Set	Reads Mapped Confidently to Probe Set	Fraction Reads in Spots Under Tissue	Median Genes per Spot	Median UMI Counts per Spot	Genes Detected
p1	49	male	I	4825	67236.18	66119.21	0.97	0.99	0.94	0.99	6218	19906	18056
p2	53	female	I	4258	75977.88	71074.36	0.85	0.99	0.99	0.94	6164	31282	18052
p3	51	female	II	4986	71070.03	70253.86	1	0.98	0.81	1	1566.5	19941	18040
p4	40	male	II	4692	74092.74	72944.5	0.94	0.99	0.98	0.99	7138.5	30512.5	18059
p5	53	female	I	4962	64385.89	63747	0.99	0.99	0.99	1	7729.5	38078.5	18047
p6	35	male	III	4990	69052.37	68482.48	1	0.99	0.98	1	5697.5	14317	18062
p7	56	female	III	4992	70420.55	69833.25	1	0.99	0.85	1	3544.5	8679.5	18072
p8	57	male	IV	4486	72496.57	71287.92	0.9	0.99	0.98	0.99	5199.5	11014	18050

Table S2: The top 50 differentially expressed genes of intra-tumor immune clusters in immune activation samples.

P1	P2	P3	P4
IGHG1	IGHG1	IGHM	IGHG1
IL32	IGKC	IGKC	IGKC
IGHG3	IGHM	CCL19	IL32
CCL19	IGHG3	IGHG1	CCL19
IGHM	CCL19	JCHAIN	IGHG3
JCHAIN	JCHAIN	IGHG3	IGHM
CXCL10	COL1A1	CYP1B1	UBD
LYZ	DCN	TRAC	CXCL9
CRP	LYZ	TRBC1	MZB1
CD74	CD44	TNFSF14	CCL5
UBD	LSP1	TRAC	TRBC2
SOD2	CORO1A	TIMP1	PSMB9
CXCL9	LUM	TSC22D3	TRAC
CTSS	COL1A2	MGP	TRBC1
SPINK1	LTB	ISLR	TNFSF14
IGLC1	CAPG	THBS2	CP
SELENOM	TIMP1	ADAMTS1	CD74
STAT1	IGHA1	NR4A1	STAT1
B2M	CD52	IL32	PLAAT4
TYMP	CD37	CD3D	TAP1
MZB1	CHI3L1	LYZ	IL32
CD68	GPNMB	LUM	CD3D
TAP1	IGLC1	CXCR4	B2M
PLAAT4	ISLR	IL7R	CD2
LAPTM5	CD74	ARID5B	IRF1
CXCL5	CXCL9	CCDC80	CTSS
LBP	MMP9	CRISPLD2	PIM2
SRGN	TRAC	SPARC	LCP1
IFI30	SFRP4	DNAJA4	NKG7
S100A8	LCP1	CCDC102B	CD3E
TMSB10	MS4A1	IGFBP5	LSP1
SGK1	IKZF1	VCAN	PLA2G2A
BIRC3	TMSB4X	CYTIP	RAC2
LRRC42	S100A6	FBLN2	DEFB1

IFITM3	MZB1	IGFBP4	GBP1
TNFSF10	TRBC2	ITGBL1	CORO1A
BCL2A1	ITGAX	IGHM	GBP2
PSMB9	ITGB2	COL14A1	TAP2
TWF2	SPI1	TGFB3	PSMB10
GSDMD	PLEKHO1	TRBC2	TYMP
MDK	COL3A1	SPARCL1	IGLC1
CD44	CTSS	IGLC1	GBP4
WFDC2	WIPF1	DCN	GBP5
IFI27	CYBB	IKZF1	TXNDC5
APOL3	VCAN	TRAC	ARHGDIB
LAP3	THBS2	CD37	EVL
TMSB4X	COTL1	PTGDS	NLRC5
ACTB	RAC2	CD44	SOD2
IDH2	CD79A	LTF	SEL1L3
EVL	IGKC	MUC6	LBP

Table S3: The top 50 differentially expressed genes of tumor area in samples with distinct immune infiltration patterns.

cluster	gene	cluster	gene
Immune activation	HSD17B13	Immune exlusion	DKK1
Immune activation	BHMT	Immune exlusion	SPP1
Immune activation	ADH4	Immune exlusion	DLK1
Immune activation	CYP8B1	Immune exlusion	GPX2
Immune activation	TAT	Immune exlusion	NQO1
Immune activation	ANO1	Immune exlusion	CRYAB
Immune activation	RDH16	Immune exlusion	AFP
Immune activation	CYP7A1	Immune exlusion	CRP
Immune activation	GLS2	Immune exlusion	PCSK1N
Immune activation	SEC14L2	Immune exlusion	SCD
Immune activation	IFI27	Immune exlusion	LCN2
Immune activation	ADH1B	Immune exlusion	AKR1B10
Immune activation	APOC4	Immune exlusion	RELN
Immune activation	GSTA2	Immune exlusion	LYZ
Immune activation	ETNPPL	Immune exlusion	CAV2
Immune activation	GPLD1	Immune exlusion	PIGR
Immune activation	MNS1	Immune exlusion	COL1A1
Immune activation	DCXR	Immune exlusion	TIMP1
Immune activation	IGHG1	Immune exlusion	LY6E
Immune activation	ALDOB	Immune exlusion	COL3A1
Immune activation	GPR88	Immune exlusion	UCHL1
Immune activation	SLC27A5	Immune exlusion	KRT23
Immune activation	PDK4	Immune exlusion	COL1A2
Immune activation	KLKB1	Immune exlusion	CTHRC1
Immune activation	GSTA1	Immune exlusion	LAPTM4B
Immune activation	TTR	Immune exlusion	MGP
Immune activation	FNDC5	Immune exlusion	TESC
Immune activation	FH	Immune exlusion	FOS
Immune activation	SLC10A1	Immune exlusion	SERPINE2
Immune activation	ACAA2	Immune exlusion	DCDC2
Immune activation	ACSL1	Immune exlusion	UGT1A9
Immune activation	PLA2G2A	Immune exlusion	CCN2
Immune activation	HMGCS2	Immune exlusion	FXYD2
Immune activation	SLPI	Immune exlusion	QSOX1
Immune activation	IGKC	Immune exlusion	MBL2

Immune activation	CYP2B6	Immune exlusion	VCAN
Immune activation	FETUB	Immune exlusion	GLUD1
Immune activation	BEX1	Immune exlusion	BGN
Immune activation	CYB5A	Immune exlusion	ABCB1
Immune activation	FMO3	Immune exlusion	SERPINE1
Immune activation	GOT2	Immune exlusion	SPINK1
Immune activation	KNG1	Immune exlusion	WNK2
Immune activation	AZGP1	Immune exlusion	CTSC
Immune activation	G0S2	Immune exlusion	MYC
Immune activation	ARG1	Immune exlusion	LUM
Immune activation	CES2	Immune exlusion	CCL20
Immune activation	TF	Immune exlusion	ANXA13
Immune activation	SLC22A7	Immune exlusion	SNORC
Immune activation	CTH	Immune exlusion	SLC9A3
Immune activation	HSD11B1	Immune exlusion	DCN
